# Infrared visualized snakes-inspired artificial vision systems with CMOS sensors-integrated upconverters

**DOI:** 10.1038/s41377-025-02001-x

**Published:** 2025-08-20

**Authors:** Ge Mu, Yangye Lin, Kerui Fu, Xin Tang

**Affiliations:** 1 School of Optics and Photonics, Beijing Institute of Technology, Beijing, 100081 China; 2 Beijing Key Laboratory for Precision Optoelectronic Measurement Instrument and Technology, Beijing, 100081 China; 3 Yangtze Delta Region Academy of Beijing Institute of Technology, Jiaxing, 314019 China

**Keywords:** Optoelectronic devices and components, Quantum dots

## Abstract

Biological visions have inspired the development of artificial vision systems with diverse visual functional traits, however, the detected wavelength is only in visible light between 0.4 and 0.78 μm, restricting their applications. Snakes generate a thermal image of animals due to pit organs for detecting and converting infrared, allowing them to accurately target predators or prey even under darkness. Inspired by natural infrared visualized snakes, we propose artificial vision systems with CMOS sensors-integrated upconverters to break visible light limitations to realize 3840 × 2160 ultra-high-resolution short-wave infrared (SWIR) and mid-wave infrared (MWIR) visualization imaging for the first time. Through colloidal quantum dot barrier heterojunction architecture design of infrared detecting units and the introduction of co-hosted emitting units, the luminance and upconversion efficiency reach up to 6388.09 cd m^−2^ and 6.41% for SWIR, 1311.64 cd m^−2^ and 4.06% for MWIR at room temperature. Our artificial vision systems broaden a wide spectrum of applications within infrared, such as night vision, agricultural science, and industry inspection, marking a significant advance in bioartificial vision.

## Abstract (web summary)

Snake-inspired artificial vision of CMOS sensors-integrated upconverters breaks visible light limitations to achieve ultra-high-resolution infrared visualization imaging.

## Graphical abstract

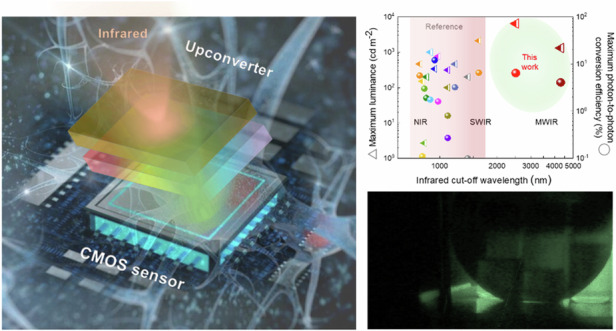

## Introduction

Biological visions have inspired the development of artificial vision systems with diverse visual functional traits that have come to the fore recently. For example, the human eye-inspired artificial visual system using a spherical biomimetic electrochemical eye with a hemispherical perovskite nanowire array retina has received great attention^1^. Besides, the eyes of animals such as aquatic^2^, feline^3^, avian^4^, and fiddler crabs^5^ have inspired artificial vision systems possessing various bionic characteristics of wide-field-of-view, superior object detection and recognition, foveated and multispectral, and panoramic visual field imaging. Despite a variety of visual features that have been realized, the detected wavelength of artificial vision systems is only in visible light between 0.4 and 0.78 μm, restricting their applications.

**Fig. 1:**
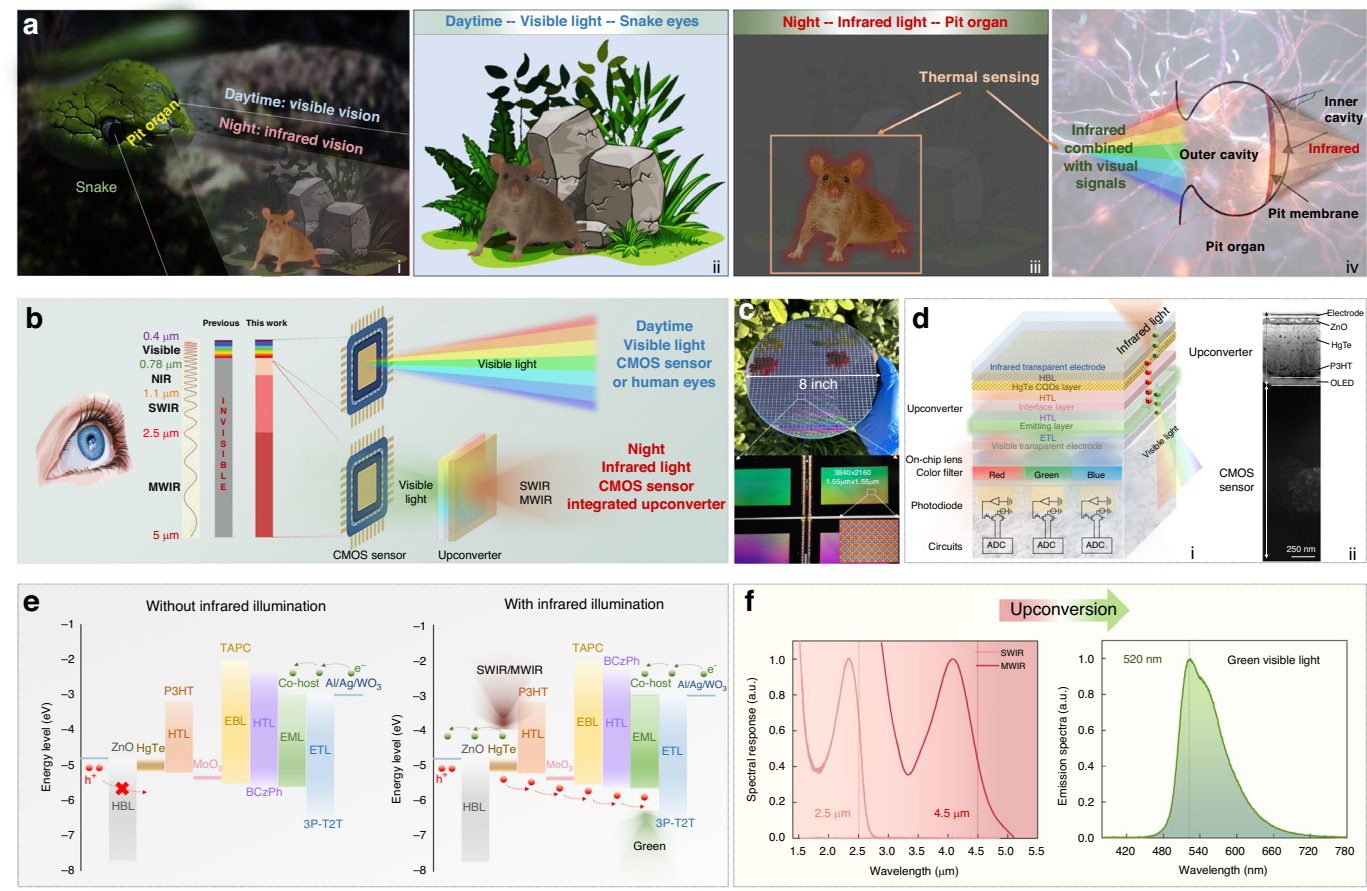
**Architecture design and operational mechanism.** **a** (i) Schematic illustration showing the snake vision systems during daytime and night. (ii) Snakes focus on their target objects through the eyes during the daytime. (iii) Snakes generate a “thermal image” of prey through the pit organ for detecting infrared radiation. (iv) Schematic of the pit organ structure. **b** Schematic illustration of the artificial vision perception through integrating the CMOS silicon sensors with infrared-to-visible upconverters enabling infrared visualization. **c** Picture of an 8-inch silicon wafer, a zoomed view of the silicon sensor, and the optical micrograph image of the pixel region. **d** (i) Schematic of the architecture and (ii) cross-sectional TEM image of CMOS sensors-integrated upconverters. **e** Energy band structure diagram of upconverters without and with infrared illumination. **f** Response and emission spectra of upconverters

Snakes focus on their target objects through the eyes during the daytime, however, in darkness, they generate a “thermal image” of prey to accurately hunt due to their unique sensory named pit organ for detecting infrared radiation^6^ (Fig. 1a i–iii). The pit membrane suspended within a hollow chamber senses infrared signals, which combined with visual information in the brain of snakes allow the snake to track the animal with great precision and speed (Fig. 1a iv). Inspired by the snake pit organ, the artificial vision systems integrate the complementary metal oxide semiconductor (CMOS) silicon sensors with infrared-to-visible upconverters (Fig. 1b). The upconverters sense low-energy incident infrared photons and convert them to higher-energy visible photons, which are then detected by silicon sensors and combined with visible information. The CMOS sensors-integrated upconverters enable the artificial vision systems to break visible light limitations to “see” naturally invisible infrared light, stimulating many important applications such as night vision, agricultural science, and industry inspection.

Recently, inorganic^7^, inorganic-organic hybrid^8^, all organic^9^, and colloidal quantum dots (CQDs)-based^10,11^ infrared-to-visible upconverters have been successively proposed. In 2020, the first solution-processed all CQDs-based upconverters with a high upconversion efficiency of 6.5% have been reported^11^. Despite the infrared detection range of CQDs-based upconverters broadening from near-infrared (NIR, 0.78–1.1 μm) to short-wave infrared (SWIR, 1.1–2.5 μm)^10,11^, the mid-wave infrared (MWIR, 3.0–5.0 μm) thermal signals still could not be upconverted to visible light. This is because, with longer detection wavelengths and narrower band gaps, the dark current originating from the thermal emission in the photodetection units dramatically rises and leads to a degraded signal-to-noise ratio operating at room temperature^12–14^. More importantly, the inhomogeneous and uncontrollable spatial doping process for CQDs homojunction infrared detecting units makes it difficult to obtain large-area upconverters, which is critical for integrating large-area CMOS sensors to realize high-quality infrared visualization imaging.

Thus, in this work, we develop mercury telluride (HgTe) CQDs barrier heterojunction architecture-based upconverters and integrate them with CMOS sensors, enabling unprecedented artificial vision perception with SWIR and MWIR visualization operating under room temperature. The barrier heterojunction architecture could eliminate the tunneling current presented in the depletion region of the typical homojunction, significantly suppressing the dark current. Besides, the inhomogeneous and uncontrollable spatial doping could be avoided for barrier heterojunction architecture-based upconverters. As a result, the upconverters exhibit excellent performance of 6388.09 cd m^−2^ luminance, 6.41% upconversion efficiency, and 38 dB linear dynamic range (*LDR*) with a cut-off wavelength of 2.5 μm and 1311.64 cd m^−2^ luminance, 4.06% upconversion efficiency, and 33 dB *LDR* with a cut-off wavelength of 4.5 μm. Inspired by the snake pit organ, the artificial vision systems with CMOS sensors-integrated upconverters realize ultra-high-resolution SWIR and MWIR visualization imaging of 3840 × 2160 array format with 1.55 μm pixel pitch for the first time.

## Results

### Architecture design and operational mechanism

Through integrating CMOS sensors with upconverters as artificial vision perception, the ultra-high-resolution of tens of millions of pixels for infrared visualization imaging could be achieved at an extremely low cost. The deposition of the upconverter on the silicon sensor array with 3840 × 2160 pixels and 1.55 μm pixel size could be conducted through wafer-scale fabrication, as shown in Fig. 1c. Figure 1d (i) displays the architecture schematic constituting an infrared-to-visible upconverter and a visible silicon CMOS sensor. The upconverter stacks of CQDs-based infrared detecting photodiode including an infrared transparent electrode, hole blocking layer (HBL), HgTe CQDs infrared sensing layer, hole transport layer (HTL), detecting/ emitting units interface layer, and organic visible light-emitting diode composed of HTL, visible light emitting layer (EML), electron transport layer (ETL), and visible transparent electrode. The visible sensor comprises on-chip lenses, color filters, photodiodes, and circuits. The cross-sectional transmission electron microscope (TEM) image of the fabricated CMOS sensors-integrated upconverters is presented displaying clear layer boundaries, as shown in Fig. 1d (ii).

The energy band structure diagram of the upconverter is presented in Fig. 1e. The zinc oxide (ZnO) layer as HBL prevents holes from electrode transporting to the visible light-emitting layer in the absence of infrared illumination. Besides, the ZnO layer and poly(3-hexylthiophene-2,5-diyl) (P3HT) layer construct an interfacial barrier to form heterojunctions for HgTe CQDs-based detecting unit that block the transport of dark current without comprising the photocarrier transport efficiency, enabling the room-temperature SWIR and MWIR detection. The molybdenum trioxide (MoO_3_) layer inserted between the detecting and emitting unit as the interface layer minimizes the contact barrier to enable the photogenerated holes to smooth transport from the detecting unit to the emitting one without lateral diffusion under infrared illumination. In addition, MoO_3_ usually as the hole injection layer for organic light-emitting diodes (OLED) improves the hole carrier injection efficiency at the interface. The 1,1-bis (di-4-tolylamino-phenyl) cyclohexane (TAPC) layer is introduced as the electron-blocking layer (EBL) to further ensure the complete suppression of photogenerated electrons tunneling. Combining the synergetic roles of the MoO_3_ interface layer, TAPC EBL, and 9,9′-diphenyl-9H,9′H-3,3′-bicarbazole (BCzPh) HTL, the photogenerated holes of HgTe CQDs under infrared illumination could efficiently unimpeded transport to the visible light emitting layer, meanwhile obstructing the photogenerated electrons tunneling. The bis (2-phenylpyridine)iridium(III)-acetylacetonate (Ir(ppy)_2_(acac)) phosphorescent material co-hosted by BCzPh HTL and 2,4,6-tris(2-(1H-pyrazol-1-yl)phenyl)-1,3,5-triazine (3P-T2T) ETL as the green light EML could reduce the gap of energy band offset and enhance the luminous efficiency. Under infrared illumination, the photogenerated holes transporting to EML combine with electrons injected from the electrode, leading to visible light emission. As a result, the upconverter successfully realizes the conversion of SWIR with a cut-off wavelength of 2.5 μm and MWIR with a cut-off wavelength of 4.5 μm to green visible light with a peak wavelength of 520 nm even under room temperature, as presented in Fig. 1f.

### Room-temperature SWIR and MWIR photodetection

**Fig. 2:**
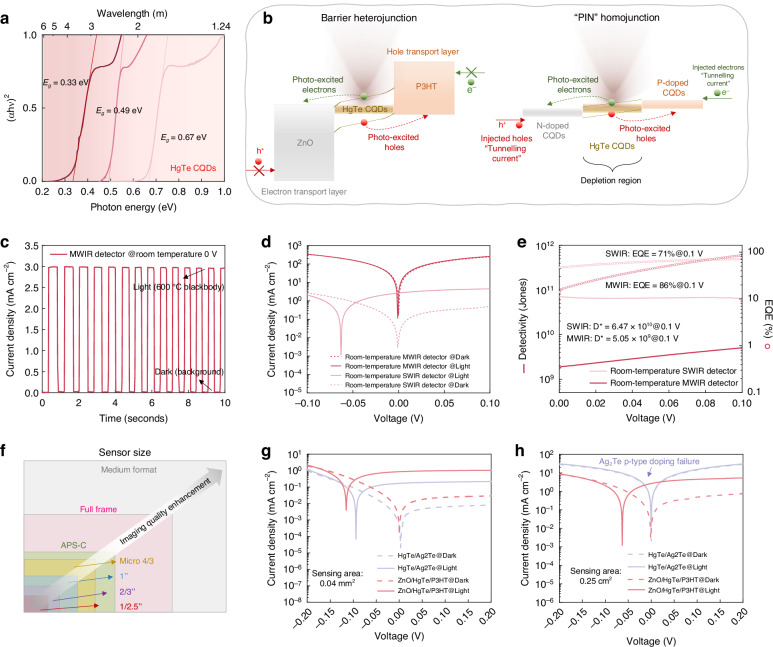
**Room-temperature SWIR and MWIR photodetection.** **a** *Tauc* plots of HgTe CQDs with an absorption wavelength from the SWIR to MWIR region. **b** Operation mechanism diagram of barrier heterojunction and “PIN” homojunction HgTe CQDs-based photovoltaic detectors. **c** Current density versus time curves of MWIR HgTe CQDs-based detectors with barrier heterojunction under room temperature at zero bias. **d** Current density versus voltage curves of SWIR and MWIR HgTe CQDs-based detectors with barrier heterojunction under room temperature. **e** Dectivity and *EQE* versus voltage curves of SWIR and MWIR HgTe CQDs-based detectors with barrier heterojunction under room temperature. **f** Schematic of the different CMOS sensor sizes. Current density versus voltage curves of homojunction HgTe/Ag_2_Te detectors and barrier heterojunction ZnO/HgTe/P3HT detectors under room temperature **g** at 0.04 mm^2^ sensing area and **h** at 0.25 cm^2^ sensing area

The HgTe CQDs with gap-less characteristics in bulk form could extend absorption wavelength to SWIR and MWIR^15–17^, thus they are chosen for our infrared sensing material. The energy bandgaps (*E*_*g*_) of HgTe CQDs could be efficiently tuned by controlling the particle size, which is shown in the Tauc plots (Fig. 2a). The operation mechanism of “PIN” homojunction and barrier heterojunction HgTe CQDs-based photovoltaic photodiodes is displayed in Fig. 2b. The gradient “PIN” homojunction structure is commonly applied in HgTe CQDs-based photovoltaic detectors^18^. However, the junction-related Shockley-read-hall (SRH) current and tunneling current that are generated in the depleted region cause the detectors to only operate under cryogenic cooling^19^. The barrier heterojunction architecture is proposed as a method to efficiently block majority-carrier dark current and without tunneling current but allows the flow of photocurrent unimpeded, which enables the photodetector to operate at high temperature^20^. The ZnO and P3HT as ETL (HBL) and HTL (EBL) for HgTe CQDs, respectively, could form efficient interfacial barriers with a large offset to block majority carriers, without affecting the photocarrier transport.

As a result, the MWIR HgTe CQDs-based detectors with barrier heterojunction exhibit strong photoresponse and high signal-to-noise ratio under 600 °C blackbody illumination even operating under room temperature at zero bias, as shown in Fig. 2c. The typical current density versus voltage curves of SWIR and MWIR HgTe CQDs-based detectors operating under room temperature are presented in Fig. 2d. The dark current density of room-temperature SWIR HgTe CQDs-based detectors is 0.48 mA cm^−2^ at 0.1 V and increases to 250 mA cm^−2^ for room-temperature MWIR ones. The dark current density under room temperature of MWIR HgTe CQDs-based detectors with barrier heterojunction is much lower than that of other reported homojunction CQDs-based detectors^21^ and epitaxial semiconductors-based detectors^22,23^. It is because the CQD energy quantization significantly reduces thermally activated carriers and band-engineered heterojunction architecture efficiently suppresses dark current.

Figure 2e shows the specific detectivity of SWIR and MWIR HgTe CQDs-based detectors with barrier heterojunction calculated by Equations S1 and S2. At 0.1 V, the noise equivalent power (*NEP*) of the SWIR and MWIR HgTe CQDs-based detectors with barrier heterojunction operating under room temperature is 7.72 × 10^−12^ and 9.91 × 10^−11 ^W, respectively. The detectivity of SWIR and MWIR HgTe CQDs-based detectors with barrier heterojunction is 6.47 × 10^10^ and 5.05 × 10^9^ Jones at 0.1 V operating under room temperature, respectively, which is more than one order of magnitude higher compared with previously reported homojunction HgTe CQDs-based detectors^13,24^. Besides, the external quantum efficiency (*EQE*, Equation S3) of the detecting units is very important, showing the photon-to-electron conversion capability^25^. The *EQE* of SWIR and MWIR HgTe CQDs-based detectors is high at 71 and 86% at 0.1 V, respectively, even when operating under room temperature, as shown in Fig. 2e. The room-temperature high-sensitivity SWIR and MWIR photodetection performance enables the upconverters to broaden the infrared region to SWIR and even MWIR.

The large area of upconverters is needed for integrating large format size CMOS image sensors that enable the single pixel to be large area improving the ability to capture photons for high-quality imaging (Fig. 2f). The premise of obtaining large-area upconverters is to fabricate large-area uniform film for infrared detecting units. The p-type doping in the HgTe CQDs is commonly acquired by introducing silver telluride (Ag_2_Te) nanocrystals under the solid-state cation exchange^15^. However, the homojunction difficulty forms at large sensing areas for HgTe CQDs-based photovoltaic detectors due to the spatial inhomogeneity and diffusion uncontrollability of the Ag^+^ ion dopants. The phenomenon is confirmed by the built-in potential of the homojunction HgTe/Ag_2_Te detectors forming in a small sensing area of 0.04 mm^2^ but disappearing in a large sensing area of 0.25 cm^2^, as shown in Fig. 2g and h. By designing band-engineered heterojunction with large band offsets ensures efficient carrier separation and minimizes recombination, the built-in potential can be amplified. The open circuit voltages of ZnO/HgTe/P3HT detectors are 115 mV for 0.04 mm^2^ sensing area and 63 mV for 0.25 cm^2^ sensing area at room temperature (Fig. 2g and h). Besides, the performance of barrier heterojunction ZnO/HgTe/P3HT detectors is remarkably enhanced compared with that of the homojunction HgTe/Ag_2_Te detectors at the small sensing area of 0.04 mm^2^, as shown in Figure S1. Furthermore, the root mean square (RMS) roughness of HgTe/P3HT heterojunction films is only 3.33 nm, which is smaller than that of HgTe/Ag_2_Te homojunction of 7.30 nm, as presented in atomic force microscope (AFM) of Figure S2. Thus, the barrier heterojunction architecture design of infrared detecting units is critical for large-area upconverters to integrate large-format CMOS image sensors.

### Transparent co-hosted visible emission

**Fig. 3:**
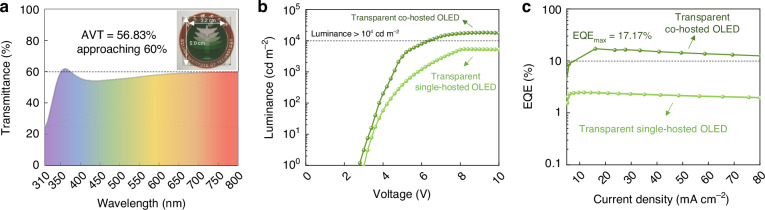
**Transparent co-hosted visible emission.** **a** Transmittance spectrum of the Al/Ag/WO_3_ visible transparent electrode. **b** Luminance versus voltage curves of transparent co-hosted and single-hosted OLEDs. **c** *EQE* versus current density curves of transparent co-hosted and single-hosted OLEDs

The visible transparent electrodes of emitting units are necessary for through visible light from the upconverters to the CMOS silicon sensors minimizing loss of visible luminance. Different visible transparent electrodes have been attempted with the transmittance spectrum shown in Figure S3. The aluminum/ silver (2 nm Al/ 8 nm Ag) thin film capping with tungsten trioxide (30 nm WO_3_) is the most transparent within the entire visible range and displays an average visible transmittance (AVT, Equation S4) approaching 60%, as shown in Fig. 3a. Note that, the performance inevitably reduces for transparent OLED with both transparent electrodes due to the leaked visible photons from the other side compared with standard one with only one transparent electrode.

To fully exploit photogenerated hole carriers from the infrared detecting units, the HTL material of BCzPh and ETL material of 3P-T2T are introduced as the co-host for phosphorescent emitting material Ir(ppy)_2_(acac). Compared with traditional single-hosted EML of only 4,4′-bis(N-carbazolyl)− 1,1′-biphenyl (CBP) as the host for Ir(ppy)_2_(acac), the transparent co-hosted OLED exhibits higher luminance (17578.49 cd m^−2^) under the same bias voltage and is above three times of single-hosted one (5234.91 cd m^−2^) at 10 V, as shown in Fig. 3b. The turn-on voltage (the required driving voltage for obtaining the luminance of 1 cd m^−2^) of the transparent co-hosted OLED is 2.7 V, which is lower compared with the single-hosted one. The current density versus voltage curve of transparent co-hosted OLED is presented in Figure S4. A saturated green light with a peak wavelength of 520 nm is shown in the electroluminescence (*EL*) spectra of transparent co-hosted OLED and the peak wavelength remains unchanged with the increase of voltage, indicating good stability in the *EL* emission (Figure S5). The *EQE* reflects the overall luminance efficiency of the OLED that could be calculated by Equation S5, showing electron-to-photon conversion capability. The transparent co-hosted OLED demonstrates a high *EQE* of 17.17% while the *EQE* of the single-hosted one is below 10%, as shown in Fig. 3c. The significantly improved performance of co-hosted OLED compared with single-hosted one is attributed to the formed barrier-free and balanced charge transportation channels in the co-hosted emission layer^9^. Therefore, the co-hosted emitting units are beneficial for the performance improvement of upconverters.

### High-performance SWIR and MWIR upconversion

Through integrating room-temperature SWIR/MWIR detecting photodiodes and transparent co-hosted visible emitting diodes, the upconverters with excellent performance were successfully fabricated. It should be noted that the performance of upconversion efficiency and luminance is ineluctably reduced more than three times due to the use of both transparent cathode and anode for upconverters instead of the standard reflective cathode^26^. While transparent upconverters are necessary for integrating with CMOS sensors for artificial vision systems.

**Fig. 4:**
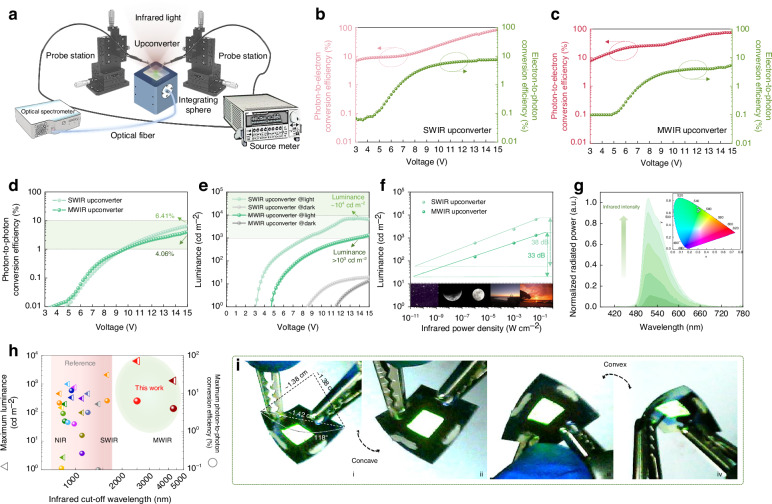
**High-performance SWIR and MWIR upconversion.** **a** Schematic diagram of the performance measurement setup of upconverters. Photon-to-electron and electron-to-photon conversion efficiency versus voltage curves of **b** SWIR-to-visible and **c** MWIR-to-visible upconverters. **d** Photon-to-photon conversion efficiency versus voltage curves of SWIR-to-visible and MWIR-to-visible upconverters. **e** Luminance versus voltage curves of SWIR-to-visible and MWIR-to-visible upconverters. **f** Luminance versus infrared power density of SWIR-to-visible and MWIR-to-visible upconverters from twilight down to starlight (10^−10^ ~ 10^−9^ W cm^–2^) conditions. **g** Emitting spectra of upconverters under different infrared intensities. Inset: *CIE* 1931 color coordinates of the visible light emitted by upconverters. **h** Performance comparison of upconverters in this work and the reported representative literature. **i** Photographs of the flexible upconverters when bent

The performance of upconverters was measured under room temperature and the schematic diagram of the setup is shown in Fig. 4a. The photon-to-electron and electron-to-photon conversion efficiency of SWIR-to-visible and MWIR-to-visible upconverters is shown in Fig. 4b, c, respectively. The photon-to-photon upconversion efficiency is an important figure of merit for evaluating upconverter performance and is calculated by Equation S6. The maximum upconversion efficiency of SWIR-to-visible and MWIR-to-visible upconverters is 6.41% and 4.06% at 15 V, respectively (Fig. 4d).

Figure 4e shows the luminance of visible light emitted by SWIR-to-visible and MWIR-to-visible upconverters versus voltage curves under dark and infrared conditions. The luminance of 6388.09 and 1311.64 cd m^−2^ is achieved at 15 V under infrared illumination of 0.05 W cm^-2^ with the turn-on voltage of 3.1 V and 4.7 V for SWIR-to-visible and MWIR-to-visible upconverters, respectively, while very faint visible light of around 10 cd m^−2^ is observed without infrared stimulus. The upconversion luminance exhibits a strong nearly linear correlation with the infrared intensity, as shown in Fig. 4f. The *LDR* of upconversion luminance with respect to the infrared power density could be calculated by Equation S7, focusing on the contrast ratio of the image quality^26^. The *LDR* of SWIR-to-visible and MWIR-to-visible upconverters is 38 dB and 33 dB at 15 V, respectively. The emitting spectra of upconverters under different infrared intensities and the corresponding Commission Internationale de l’Eclairage (*CIE*) chromaticity coordinate are shown in Fig. 4g. The performance comparison of transparent upconverters in this work and the reported representative literature (with standard reflective electrodes) is summarized in Fig. 4h and Table S1. This is the first time to realize MWIR upconversion to visible light with a detection cut-off wavelength of 4.5 μm, while the detection region of most current upconverters is still limited in NIR around 1 μm. The luminance and upconversion efficiency of SWIR-to-visible and MWIR-to-visible upconverters in this work are competitive with previously reported NIR ones^27–35^. The broadening of the infrared detection band of upconverters from NIR to SWIR and MWIR is very important for artificial vision systems to stimulate many new application fields such as industry inspection, food safety, gas sensing, autonomous driving, and night vision imaging.

To mimic and implement key structural and functional features of the biological vision systems, a curved form in the imaging system is necessary^36^. The flexible upconverters exhibit excellent bendability whether concave or convex, as displayed in Fig. 4i. Under considerable bending with a bending angle of 62°, the flexible upconverters enable direct real-time infrared visualization to emit saturated green light under infrared illumination. The flexible, lightweight, low-cost, and high-performance upconverters as curved image sensors could implement an artificial vision device.

### Artificial vision systems for ultra-high-resolution infrared imaging

**Fig. 5:**
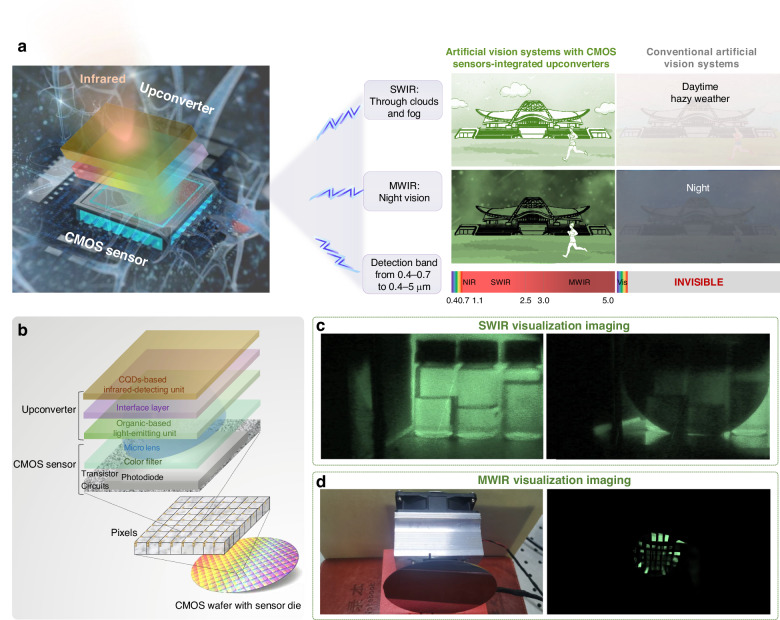
**Artificial vision systems for ultra-high-resolution infrared imaging**. **a** Imaging comparison diagram of artificial vision systems with CMOS sensors-integrated upconverters and conventional ones. **b** Integration process diagram of upconverters into CMOS sensors. Images of **c** vials of different chemical solvents and a silicon wafer and **d** the array of light sources and a silicon wafer captured by artificial vision systems with CMOS sensors-integrated upconverters

The artificial vision systems inspired by the natural snakes can be developed by integrating high-performance upconverters with CMOS image sensors (Fig. 5a). The upconverters inspired by the pit membrane of snakes’ pit organs enable the detection of infrared light and convert it to visible signals. The integrated CMOS sensors sense the converted visible light from infrared analogous to the thermal image processing and generation process of snakes. Conventional artificial vision systems are confined within visible regions. Under the influence of extreme weather such as haze, conventional artificial vision systems cannot see objects behind the smoke because the visible light is reflected and cannot pass through small particles such as fog and smoke. However, artificial vision systems with CMOS sensors-integrated upconverters realizing infrared imaging dramatically improve the adaptability to climate conditions due to SWIR’s strong ability to penetrate fog, mist, and smoke, and long effective detection distance. Besides, the integrated sensor has the ability to see in the dark because of the broadened detection region from visible to MWIR, which could capture thermal infrared radiation by objects. The detection spectral range of the artificial vision systems with CMOS sensors-integrated upconverters is expanded by 14 times, from 0.4 ~ 0.7 μm to 0.4 ~ 4.5 μm. As a result, artificial vision systems with CMOS sensors-integrated upconverters enable high-resolution imaging in all weather, whether day or night, regardless of extreme weather. Figure 5b displays the integration of the upconverter constituting CQDs-based infrared heterojunction photodiodes, interface layers, and energy band-matched organic-based light-emitting diodes into wafer-level CMOS silicon sensor array comprising micro-lenses, color filters, photodiodes, transistors, and circuits.

The resolution and format area of infrared imaging depends on the chosen CMOS image sensors on the premise of achieving high-performance upconverters. In this work, 3840 × 2160 pixels with 1.55 μm pixel size CMOS image sensors are used to integrate with upconverters. High-resolution SWIR and MWIR imaging with a frame rate of 120 Hz is achieved captured by artificial vision systems with CMOS sensors-integrated upconverters. The opaque silicon wafer blocking the vials in visible light (Figure S6) becomes transparent, indicating the SWIR imaging capability, as shown in Fig. 5c. Besides, thermal imaging is demonstrated for the array of light sources clearly observed through the silicon wafer (Fig. 5d).

## Discussion

We develop artificial vision systems with CMOS sensors-integrated upconverters inspired by natural infrared visualized snakes and demonstrate the application of 3840 × 2160 ultra-high-resolution SWIR and MWIR visualization imaging under room temperature. Due to the barrier heterojunction configuration for CQDs-based infrared detecting units, the efficiently suppressed dark current without affecting the photocarrier transport efficiency enables the upconverters to room-temperature high-sensitivity SWIR and MWIR sensing. Besides, the co-hosted emitting units with high luminous efficiency due to the formed balanced charge transportation channels in emitting layers are beneficial for the performance improvement of upconverters. As a result, the performance such as luminance (6388.09 cd m^−2^ for SWIR, 1311.64 cd m^–2^ for MWIR), upconversion efficiency (6.41% for SWIR, 4.06% for MWIR), and *LDR* (38 dB for SWIR, 33 dB for MWIR) of upconverters remain higher than previously reported NIR ones. The extended artificial vision into infrared range could operate in all weather, whether day or night, regardless of extreme weather, and be of use in new fields such as industry inspection, food safety, gas sensing, agricultural science, and autonomous driving.

## Materials and methods

### Material synthesis

The synthesis of SWIR and MWIR HgTe CQDs is similar to previous reports^37^. Mercury chloride (HgCl_2_, Strem Chemicals, 99%, 0.4 mmol) was dissolved in 16 ml of oleylamine (OAM, Aladdin) in a 30 ml glass vial at 100 °C for 1 h with stirring in the glove box. The temperature was then adjusted to the reaction temperature and stabilized for 30 min. Tellurium power (Te, Sigma-Aldrich, 99.999%) in trioctylphosphine (TOP, Sigma-Aldrich, 97%) solution (1 M, 0.4 ml) was rapidly injected. The clear solution immediately turned black. The reaction temperature and reaction time depend on the target size for the HgTe CQDs. The reaction was quenched by injecting a solution of 3 ml dodecanethiol (DDT, Sigma-Aldrich, 98%) and 0.4 ml TOP in 16 ml tetrachloroethylene (TCE, Aladdin, 98%). After quenching, the vial was quickly removed from the glove box and cooled down. The solution was precipitated with an equal volume of isopropanol (IPA) and centrifuged at 5000 rpm for 5 min. Finally, the precipitate was resuspended in solvents avoiding disruption of underlying layers when fabricating devices.

### Device fabrication

The upconverters were fabricated on the CMOS silicon sensors, which are commercially sourced. The visible emitting unit of Al (2 nm)-Ag (8 nm)-WO_3_ (30 nm)/3P-T2T (25 nm)/ BCzPh: 3P-T2T: Ir(ppy)_2_(acac) (1:1:8% 30 nm)/ BCzPh (30 nm)/ TAPC (30 nm) was evaporated at 5 × 10^−5^ mba. The detecting/ emitting units interface layer of MoO_3_ (3 nm) was deposited by thermal evaporation. The infrared detecting unit of P3HT (10 mg ml^−1^), HgTe CQDs, and ZnO solution were spin-coated at 3000 rpm for 30 s. The infrared transparent electrode such as ITO was deposited using an Angstrom Engineering deposition system. HgTe contains toxic heavy metals and careful handling is required during the process of material synthesis and device preparation.

### Characterization

The cross-section of devices is sliced up by a focused ion beam electron beam double beam scanning electron microscope (Helios G4 UC) and cross-sectional TEM images were obtained by field emission transmission electron microscope (Talos F200X G2). The absorption spectra of HgTe CQDs were characterized by a Fourier transform spectrometer (Nicolet™ iS20 FTIR Spectrometer). The current versus time or voltage curves of detectors were measured using the source meter (Keithley 2602B) with a calibrated blackbody as the light source. The luminance and efficiency of visible light emitted by OLEDs and upconverters were measured by the spectrometer with an integration sphere.

## Supplementary information

Supplementary Information

## Data Availability

The data that support the findings of this study are available from the corresponding author upon reasonable request.
