# Supplementary material for: Infrared visualized snakes-inspired artificial vision systems with CMOS sensors-integrated upconverters
Source: Light Sci Appl. 2025 Aug 20;14:282. doi: 10.1038/s41377-025-02001-x (PMC12368111; doi:10.1038/s41377-025-02001-x)
Supplement: Supplementary file 1 — Supplementary Information [file 41377_2025_2001_MOESM1_ESM.docx]

**Supplementary Information for**

Infrared visualized snakes-inspired artificial vision systems with CMOS sensors-integrated upconverters

Ge Mu^1*^, Yangye Lin^1^, Kerui Fu^1^, Xin Tang^1,2,3*^

^1^School of Optics and Photonics, Beijing Institute of Technology, Beijing 100081, China

^2^Beijing Key Laboratory for Precision Optoelectronic Measurement Instrument and Technology, Beijing, 100081, China

^3^Yangtze Delta Region Academy of Beijing Institute of Technology, Jiaxing, 314019, China

^*^Corresponding author. [xintang@bit.edu.cn](mailto:xintang@bit.edu.cn) (X. T.), gemu@bit.edu.cn (G.M.)

**Section S1. Room-temperature SWIR and MWIR photodetection**

The performance of SWIR and MWIR HgTe CQDs-based detectors is characterized under 600 ℃ blackbody. The “light” current is collected with the 600 ℃ blackbody radiation. The “dark” current is collected with room-temperature background radiation.

The responsivity (*ℜ*, units of *A W^-1^*) of detectors can be obtained by Equation S1:

$\Re=\frac{I_{ph}}{P}$ (S1)

where *I_ph_* is the photocurrent (*A*) and *P* is the radiation optical power (*W*).

The detectivity (*D^*^*, units of *cm⋅Hz^1/2^⋅W^-1^* or Jones) of detectors is a measure of the signal-to-noise ratio for a given incident power and normalized to the detector area shown in Equation S2:

$D^{*}=\frac{\sqrt{A\cdot\Delta f}}{NEP}=\frac{\sqrt{A\cdot\Delta f}}{I_{n}}\Re$ (S2)

where *A* is the area of the detector (*cm^2^*), *Δf* is the bandwidth (*Hz*), *NEP* is the noise equivalent power (*W*), *I_n_* is the RMS noise current (*A*), and ℜ is the responsivity (*A W^-1^*).

The external quantum efficiency (*EQE*, units of %) of detectors is the ratio of the number of carriers collected by the detectors to the number of incident photons given by Equation S3:

$EQE=\frac{1.24}{\lambda}\mathfrak{R}$ (S3)

where *λ* is the wavelength of the infrared light with units of μm, and *ℜ* is the responsivity.

**
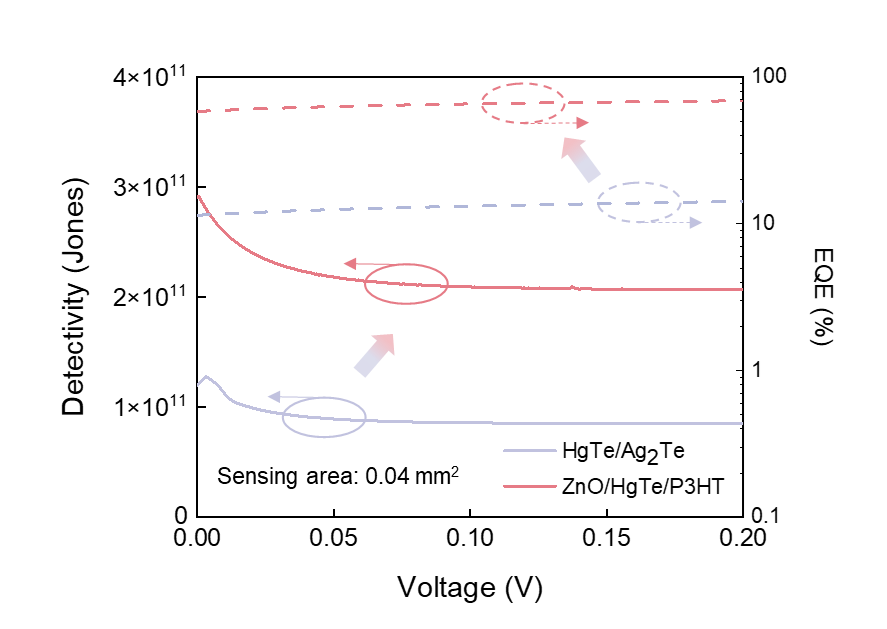
**

**Figure S1.** Dectivity and *EQE* versus voltage curves of homojunction HgTe/Ag_2_Te detectors and barrier heterojunction ZnO/HgTe/P3HT detectors at 0.04 mm^2^ sensing area under room temperature.

**
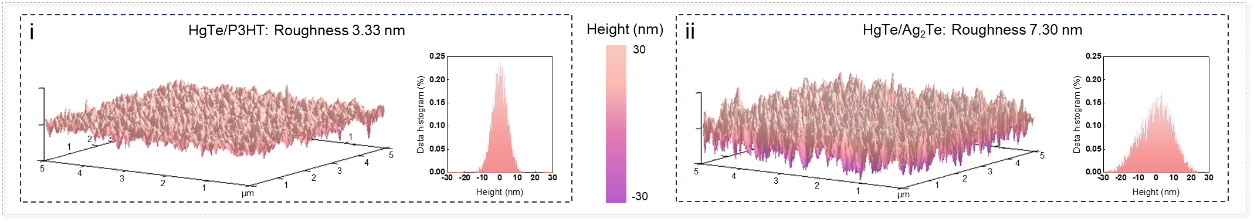
**

**Figure S2.** Atomic force microscope (AFM) and corresponding histogram of **i.** HgTe/P3HT heterojunction and **ii.** HgTe/Ag_2_Te homojunction.

**Section S2. Transparent co-hosted visible emission**

**
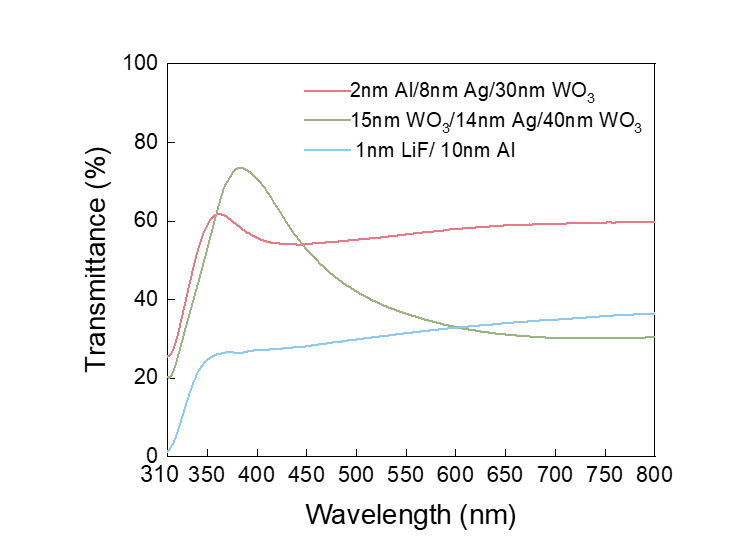
**

**Figure S3.** Transmittance spectrum of the different visible transparent electrodes.

The average visible transmittance (*AVT*) can be calculated by Equation S4:

$\text{AVT=}\frac{\int T(\lambda)P(\lambda)S(\lambda)d(\lambda)}{\int P(\lambda)S(\lambda)d(\lambda)}$ (S4)

where *T*, *P*, *S*, and *λ* are the transmission spectrum, the photopic response of human eyes, solar photon flux (AM 1.5 G), and the wavelength of light, respectively.


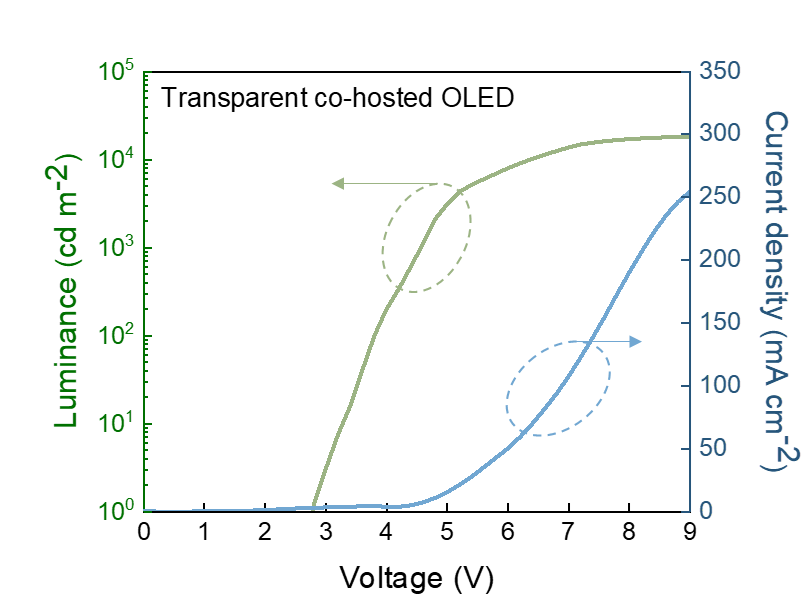


**Figure S4.** Luminance and current density versus voltage curves of transparent co-hosted OLED.


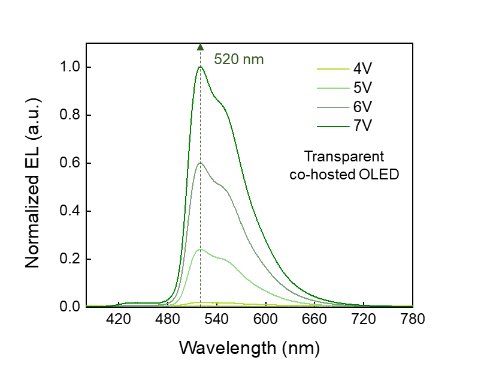


**Figure S5.** Electroluminescence (*EL*) spectra of transparent co-hosted OLED under different bias voltages.

The *EQE* (units of %) of organic light-emitting diodes (OLED) is the ratio of the number of emitted photons to the number of injected electrons given by Equation S5:

 (S5)

where *e* is the electron charge, *h* is the Planck constant, *c* is the velocity of light, *K_m_* is the maximum luminous efficacy, *J* is the current density, *L* is the luminance, *λ* is the wavelength, *I*(*λ*) is the relative *EL* intensity at a wavelength *λ* determined by the *EL* spectra, *V*(*λ*) is the normalized photonic spectral response function.

**Section S3.** **High-performance SWIR and MWIR upconversion**

The photon-to-photon upconversion efficiency (units of %) is calculated by the number of visible light photons emitted by the upconverter over the number of incident infrared photons given by Equation S6:

$\eta_{p-p}=\frac{N_{e}}{N_{i}}=\frac{P_{e}/\frac{hc}{\lambda_{e}}}{P_{i}/\frac{hc}{\lambda_{i}}}$ (S6)

where *N_e_* and *N_i_* are the number of emitted visible photons and incident infrared photons, respectively; *P_e_* and *P_i_* refer to the power of the emitted visible photons and incident infrared photons, respectively; *λ_e_* and *λ_i_* are the wavelengths of the emitted visible light and incident infrared light, respectively; *h* is Planck constant; *c* is the speed of light.

The linear dynamic range (*LDR*, units of *dB*) of upconverters could be calculated given by Equation S7^1^:

$LDR=20\log(\frac{L_{\max}}{L_{\min}}\text{）}$ (S7)

where *L_max_* and *L_min_* are the linear luminance corresponding to the upper and lower limit of incident infrared power density.

**Table S1.** Comparison of performance for infrared-to-visible upconverters.

| Upconverters | Detection/ emission wavelengths (nm) | Material | Luminance (cd m^-2^) | Photon-to-photon conversion efficiency (%) | Linear dynamic range (*dB*) | Infrared imaging resolution |
| --- | --- | --- | --- | --- | --- | --- |
| NIR-to-green devices^2^ | 785/ green 520 | PD: PM6:Y6; LED: PQDs | 463.9 @12 V @1 mW on the active area | 5.68@12 V | - | - |
| NIR-to-red or yellow devices^3^ | 810/ red 630, yellow 590 | PD: GaAs; LED: AlGaInP | - | 1.5 | - | - |
| NIR-to-blue, green, red or white devices^4^ | 810/ blue 480, green 512, red 584 and white 577 | PD: ING-T-DPP, PC_61_BM; LED: TADF | 150 @10 V @100 mW cm^-2^ | 0.11 @10 V | - | - |
| NIR-to-green devices^5^ | 830/ green 520 | PD: FAPbI_3_ perovskite; LED: CBP | 2.7 @5 V @354 μW cm^-2^ | 3 @5 V | 24 | - |
| NIR-to-green devices^6^ | 850/ green 520 | PD: DPP–DTT:CO*_i_*8DFIC; LED: CsPbBr_3_ perovskite | 200 @8 V @5 mW cm^-2^ | 1.9 @6 V | - | - |
| NIR-to-green devices^7^ | 860 or 890/ green 530 | PD: DPP-DTT:COi8DFIC or DPP-DTT:Y6 LED: Tm3PyBPZ | ~1000@8 V @10 mW cm^-2^ | 1.75 or 1.55@8 V | - | - |
| NIR-to-green devices^8^ | 940/ green 523 | PD: PTB7-Th; LED: BCzPh:CN-T2T:Ir(ppy)_2_(acac) (1:1:8%) | 337.2 @8 V @1 mW cm^-2^ | 12.56 @8 V | 80 | - |
| NIR-to-yellow devices^9^ | 980/ yellow 575 | PD: SQ-880:PCBM; LED: fluorescent poly(para-phenyle-ne vinylene) copolymer | 760 @7.5 V @49 mW cm^-2^ | 1.6 @12 V | - | - |
| NIR-to-green devices^10^ | 1100/ green 520 | PD: SQ-880: PCBM; LED: Alq_3_ | 313 @12 V @49 mW cm^-2^ | 0.27 @12 V | - | - |
| NIR-to-green devices^11^ | 1100/ green 520 | PD: PBDTT-BTQ; LED: 9% Ir(ppy)_3_ doped CBP | >100 @12 V | 0.8 @9.5 V | - | - |
| NIR-to-red devices^12^ | 1200/ red | PD: PbS CQDs; LED: CdSe CQDs | 460 @30 V @100 mW cm^-2^ | 3.2 @30 V | - | - |
| SWIR (1.4 μm)-to-green devices^13^ | 1400/ green 516 | PD: DPPDTT: SWIR dye blend layer; LED: CsPbBr_3_ perovskite | 200 @14 V @32 mW cm^-2^ | 0.1 @14 V | - | - |
| SWIR (1.6 μm) -to-green devices^14^ | 1600/ green 525 | PD: PbS QDs; LED: CdSe/ ZnS QDs | 2100 @11 V @10 mW cm^-2^ | 6.5 @10 V | - | Spatial resolution exceeds 16 line pair/mm |
| This work: Transparent SWIR (2.5 μm) or MWIR (4.5 μm)-to-green devices | 2500 or 4500/ green 520 | PD: HgTe CQDs; LED: BCzPh: 3P-T2T: Ir(ppy)_2_(acac) (1:1:8%) | SWIR-green: 6388.09 @15 V @0.05 W cm^-2^; MWIR-green: 1311.64 @15 V @0.05 W cm^-2^ | SWIR-green: 6.41 @15 V; MWIR-green: 4.06 @15 V | SWIR-green: 38 @15 V; MWIR-green: 33 @15 V | 3840×2160 pixels with 1.55 μm pixel size |

**Section S4. Artificial vision systems for ultra-high-resolution infrared imaging**


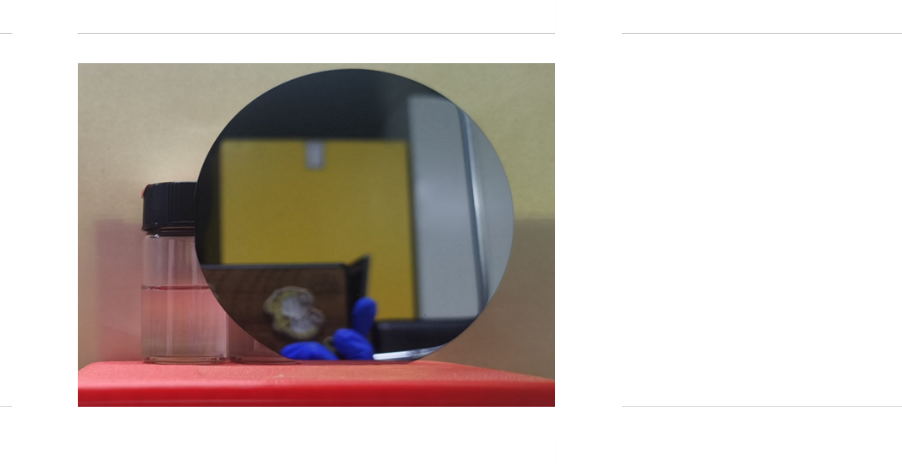


**Figure S6.** Scene of the imaging process.

The silicon-based CMOS substrate with moderate thermal conductivity of our CMOS sensors-integrated upconverters could as a heat sink to partly dissipate localized heat generated in the upconverters without significant temperature accumulation. We monitored the device temperature using infrared thermal imaging during continuous operation (3 hours at 15 V), as shown in **Figure S7**. The maximum temperature rise observed was <4 ℃, confirming negligible thermal accumulation. The upconverter performance testing and imaging of **Figures 4** and **5** were performed at continuous operating voltages. The minor temperature rise does not compromise imaging performance.

*
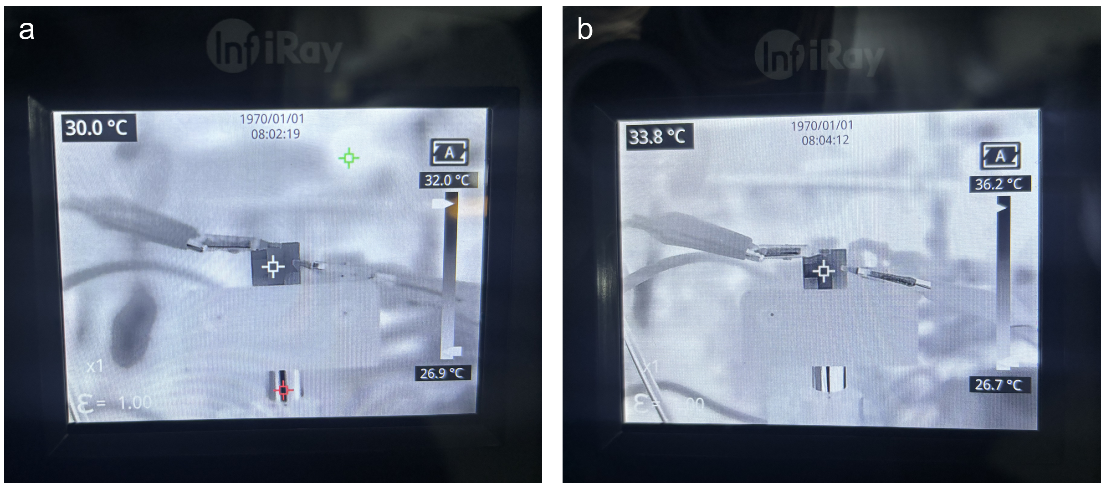
*

**Figure S7.** The device temperature using infrared thermal imaging **a.** just starting to work at 15 V and **b.** continuous operation for 3 hours at 15 V.

**Section S5. Prospects for upconverters**

Three key aspects could be optimized in the future:

(1) Infrared Detecting Unit: By designing band-engineered heterojunction with large band offsets and optimizing the energy alignment between the hole-blocking layer and hole transport layer ensures efficient carrier separation and minimizes recombination, the built-in potential can be amplified. Surface ligand engineering reduces trap states in HgTe CQDs, enhancing carrier mobility and collection efficiency. Leveraging avalanche multiplication in photodetector under strong built-in fields can amplify photocurrent, increasing the open-circuit voltage. Stacking multiple photovoltaic units in series can cumulatively increase the total open-circuit voltage to reach the turn-on voltage of the light-emitting unit.

(2) Visible Emitting Unit: Using highly efficient co-host systems achieves balanced charge transport, enabling high emission efficiency at a low driving voltage of the infrared detecting unit. Explore low-dimensional materials such as perovskite and quantum dots for ultra-low turn-on voltage emitters. The reduction of the turn-on voltage of the visible emitting unit could lower the threshold for self-driven emission.

(3) Detector/Emitter Interface: Introducing an interlayer between the detector and emitter minimizes contact resistance and improves photogenerated carriers’ transport efficiency from the detector to the emitter. Balancing the cumulative open-circuit voltage of stacked detectors with the turn-on voltage of emitters requires precise material and device engineering.

(4) Optical Resonators Design: Integrating distributed Bragg reflectors (DBRs) or plasmonic nanostructures into the detector layer can amplify infrared absorption and photocarrier generation, improving signal-to-noise ratio under weak illumination.

**References**

1. Shih, C. J. et al. Transparent organic upconversion device targeting high- grade infrared visual image. *Nano Energy* **86**, 106043 (2021).
2. Chen, L. C. et al. High-leakage-resistance and low-turn-on-voltage upconversion devices based on perovskite quantum dots. *Advanced Functional Materials* **2309589**, 1–10 (2023).
3. Ding, H. et al. Microscale optoelectronic infrared-to-visible upconversion devices and their use as injectable light sources. *Proceedings of the National Academy of Sciences of the United States of America* **115**, 6632-6637 (2018).
4. Tachibana, H. et al. Tunable full-color electroluminescence from all-organic optical upconversion devices by near-infrared sensing. *ACS Photonics* **4**, 223-227 (2017).
5. Yu, B. H. et al. Sub-band gap turn-on near-infrared-to-visible up-conversion device enabled by an organic-inorganic hybrid perovskite photovoltaic absorber. *ACS Applied Materials & Interfaces* **10**, 15920-15925 (2018).
6. Li, N. et al. NIR to visible light upconversion devices comprising an nir charge generation layer and a perovskite emitter. *Advanced Optical Materials* **6**, 1801084 (2018).
7. Li, N. et al. Wavelength-selective near-infrared organic upconversion detectors for miniaturized light detection and visualization. *Advanced Functional Materials* **2411626**, 1–9 (2024).
8. Shih, C. J. et al. Transparent organic upconversion devices displaying high-resolution, single-pixel, low-power infrared images perceived by human vision. *Science Advances* **9**, eadd7526 (2023).
9. Strassel, K. et al. Solution-processed organic optical upconversion device. *ACS Applied Materials & Interfaces* **11**, 23428-23435 (2019).
10. Strassel, K. et al. Squaraine dye for a visibly transparent all-organic optical upconversion device with sensitivity at 1000 nm. *ACS Applied Materials & Interfaces* **10**, 11063-11069 (2018).
11. Yeddu, V. et al. Low-band-gap polymer-based infrared-to-visible upconversion organic light-emitting diodes with infrared sensitivity up to 1.1 μm. *ACS Photonics* **6**, 2368-2374 (2019).
12. Kwon, T. H. et al. Quantum dot-based three-stack tandem near-infrared-to-visible optoelectric upconversion devices. *ACS Nano* **18**, 21957-21965 (2024).
13. Li, N. et al. SWIR photodetection and visualization realized by incorporating an organic SWIR sensitive bulk heterojunction. *Advanced Science* **7**, 2000444 (2020).
14. Zhou, W. J. et al. Solution-processed upconversion photodetectors based on quantum dots. *Nature Electronics* **3**, 251-258 (2020).
